# *Gongjin-Dan* Enhances Neurite Outgrowth of Cortical Neuron by Ameliorating H_2_O_2_-Induced Oxidative Damage via Sirtuin1 Signaling Pathway

**DOI:** 10.3390/nu13124290

**Published:** 2021-11-27

**Authors:** Hyunseong Kim, Wanjin Jeon, Jinyoung Hong, Junseon Lee, Changhwan Yeo, Yoonjae Lee, Seungho Baek, Inhyuk Ha

**Affiliations:** 1 Jaseng Spine and Joint Research Institute, Jaseng Medical Foundation, Seoul 135-896, Korea; biology4005@gmail.com (H.K.); poghkl@gmail.com (W.J.); vrt3757@gmail.com (J.H.); excikind@gmail.com (J.L.); duelf12@gmail.com (C.Y.); goodsmile8119@gmail.com (Y.L.); 2 College of Korean Medicine, Dongguk University, 32 Dongguk-ro, Ilsandong-gu, Goyang-si 10326, Korea; Baekone99@gmail.com

**Keywords:** *Gongjin-dan*, hydrogen peroxide, cortical neuron, Sirtuin1, nerve regeneration, neuroprotection, synapse, nerve growth factor, brain-derived neurotrophic factor

## Abstract

*Gongjin-dan* (GJD) is a multiherbal formula produced from 10 medicinal herbs and has been traditonally used as an oriental medicine to treat cardiovascular diseases, alcoholic hepatitis, mild dementia, and anemia. Additionally, increasing evidence suggests that GJD exerts neuroprotective effects by suppressing inflammation and oxidative stress-induced events to prevent neurological diseases. However, the mechanism by which GJD prevents oxidative stress-induced neuronal injury in a mature neuron remains unknown. Here, we examined the preventive effect and mechanism of GJD on primary cortical neurons exposed to hydrogen peroxide (H_2_O_2_). In the neuroprotection signaling pathway, Sirtuin1 is involved in neuroprotective action as a therapeutic target for neurological diseases. After pre-treatment with GJD at three concentrations (10, 25, and 50 µg/mL) and stimulation by H_2_O_2_ (30 µM) for 24 h, the influence of GJD on Sirtuin1 activation was assessed using immunocytochemistry, real-time PCR, western blotting, and flow cytometry. GJD effectively ameliorated H_2_O_2_-induced neuronal death against oxidative damage through Sirtuin1 activation. In addition, GJD-induced Sirtuin1 activation accelerated elongation of new axons and formation of synapses via increased expression of nerve growth factor and brain-derived neurotrophic factor, as well as regeneration-related genes. Thus, GJD shows potential for preventing neurological diseases via Sirtuin1 activation.

## 1. Introduction

Oxidative stress is a pathological hallmark of regenerative failure resulting from an oxidative imbalance between cellular oxidant and antioxidant defense in the nervous system [1,2]. One of the major reasons for being worsened with diseases progression is antioxidant system depletion, which induces the degradation of almost all cell contents including the lipid membrane, protein, and DNA [3]. Accumulation of ROS, such as hydrogen peroxide (H_2_O_2_), superoxide anion (O_2_^−^), and hydroxyl radical (HO·), that exceeds the capacity of the antioxidant system results in neuronal death and functional impairment [4]. Increasing evidence suggests a strong relationship between oxidative stress and neurological diseases, such as Alzheimer’s disease and Parkinson’s disease [5,6]. In addition, oxidative stress contributes to the pathogeneses of secondary damage after traumatic injuries to the spinal cord and brain [7,8]. Therefore, the regulation of oxidative stress has been suggested for resolving neurological diseases. In recent years, natural substances that have been empirically verified as safe and effective have been widely studied. *Gongjin-dan* (GJD) is a multi-herbal drug made by adding various herbal medicines based on *Musk*, *Cervi Pantiorichum*, *Angelicae Radix*, and *Cornus officinalis* [9]. Representatively, *Yookgong-dan* has been developed based on the GJD combination with *Yukmijihwan* [10,11]. GJD is most frequently used clinically in Korea and China as an anti-fatigue agent, particularly under conditions of sleep deprivation, and has been used as an anti-aging agent for several hundreds of years [12,13].

Experimental evidence has shown that GJD has a various pharmacological effects, such as anti-oxidative, anti-inflammatory, hepatoprotective, neuroprotective, immunological, and reproductive recovery activities and causes few side-effects [14,15,16,17,18]. Specifically, previous studies revealed that GJD may exert neuroprotective effects by enhancing memory and learning via upregulation of neurotrophic factors in neuronal-like cell lines, such as PC12 and HT22 cells [16,17], and in animal models of memory loss or stroke [18]. However, only the involvement of inflammatory cytokines and growth factors has been confirmed in the mechanism of GJD action. In addition, previous studies used several neuronal cell lines to evaluate GJD’s neuroprotective effects, which do not reflect the actual central nervous system, as mature central mamalian neurons cannot repair themselves because they lack the ability to reform new neuronal populations after injury. Therefore, the mechanisms involved in the therapeutic effect of GJD should be examined in mature central nervous system neurons. Increasing evidence supports that oxidative stress may be reduced by the activation of Sirtuin1 (Sirt1), an NAD^+^-dependent protein deacetylase, in neurological diseases [19,20,21,22]. Upregulation of Sirt1 can contribute to maintaining the antioxidant capacity via the Forkhead box protein pathways. Moreover, Sirt1 regulates ROS production by modulating peroxisome proliferator-activated receptor-1α activity, which is a major activator of mitochondrial biogenesis [23]. Recent studies also demonstrated that increased levels of ROS can control Sirt1 activity both directly and indirectly [24].

Based on these previous studies, three doses of GJD were applied to the cortical neurons with H_2_O_2_ exposure and it was assessed whether GJD enhances the neuroprotective effect against oxidative damage and neuronal death in mature cortical neurons. Specifically, our findings highlight a mechanism by which GJD may prevent neurological diseases by activating the Sirt1 signaling pathway.

## 2. Materials and Methods

### 2.1. Primary Cortical Neurons

Sprague–Dawley rats were used and permission processed with the Jaseng Animal Care and Use Committee (JSR-2021-07-003-A). The 17-day rat embryos (Daehan Bio Link, Chungbuk, Korea) were used for culturing the cortical neurons as previously described [25]. Briefly, the embryos were seperated from a pregnant rat and placed in a Petri dish containing Hank’s balanced salt solution (HBSS; Gibco BRL, Grand Island, NY, USA) on ice. Embryos were dosally positioned for removing the skin and skull using fine forceps. The cerebral cortex was divided by hemisphere in Hank’s balanced salt solution, and the meninges were manually removed. The tissues were dissociated using a neural tissue dissociation kit (Miltenyi, Bergisch Gladbach Bergisch, Germany) according to the manufacture’s protocol. Subsequently, the tissues were centrifuged at 2000 rpm for 3 min. Cells were triturated in neurobasal medium (Gibco BRL) with penicillin/streptomycin (1:100, Gibco BRL), B27 (1:50, Gibco BRL), and Gluta-MAX (1:100, Gibco BRL). The plate coatings were performed using poly-D-lysine (20 mg/mL. Gibco BRL) overnight and laminin (10 µg/mL, Sigma-Aldrich, St. Louis, MO, USA) for 2 h at 4 °C, and then seeded onto coated plates.

### 2.2. Preparation of Gongjin-dan (GJD)

GJD was prepared as a mixture of *Rehmannia glutinosa, Cervi pantiorichum, Angelicae Radix, Dioscorea polystachya, Cornus officinalis, Wolfiporia extensa, Alisma canaliculatum, Moutan Cortex* Radicis, *Musk*, and *Aquilaria agallocha* Roxburgh. GJD was dried for 24 h at 70 °C in a dryer and then finely pulverized using a grinder. The mixed powder was dissolved in 1 mL phosphate-buffered saline (PBS) for preparation the 10 mg/mL stock solution. The solution was filtered with a 0.45 μm polytetrafluoroethylene syringe filter (Advantec Co., Tokyo, Japan).

### 2.3. GJD Pretreatment and H_2_O_2_-Induced Neuronal Injury

**Scheme 1:**
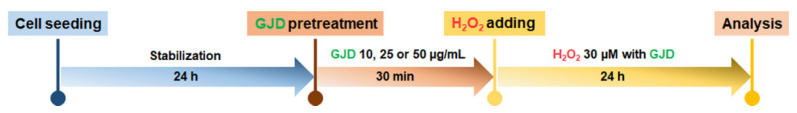
Schematic procedures in *Gongjin-dan* (GJD)-pretreated and H_2_O_2_ condition.

The seeded cells were cultured in neuron medium for 1 day, and then the medium was replaced with 10, 25, or 50 µg/mL GJD-containing medium. After 30 min of incubation to allow for the preventive effect of GJD, H_2_O_2_ was added to the medium at a concentration of 30 µM. The cells were then incubated for 24 h in the presence of both GJD and H_2_O_2_. The timeline of the experiment is shown in Scheme 1.

### 2.4. Neuronal Viability Assays

First, the Cell Counting Kit-8 (CCK-8; Dojindo, Kumamoto, Japan) was used for confirmation of the cell viability after GJD pretreatment at various doses of 1, 10, 25, 50, and 100 µg/mL with or without H_2_O_2_ exposure. CCK-8 reagent (10% of total volume) was treated and incubated for 4 h at 37 °C, and then absorbance measurement at 450 nm was analyzed using a microplate reader (Epoch, BioteK, Winooski, VT, USA). Live and dead cells were confirmed by a live/dead cell imaging kit (Thermo Fisher Scientific, Waltham, MA, USA) according to the manufacturer’s instructions. The cells were treated with live dead assay staining solution (100 µL) for 15 min at 37 °C. To quantify the number of live and dead cell, 10 images per group were randomly captured at 10× objective using a confocal microscope (Eclipse C2 Plus, Minato, Tokyo, Nikon, Japan).

### 2.5. Immunocytochemistry

The antioxidant neuroprotective effect of GJD was assessed by immunocytochemistry in the H_2_O_2_-activated cortical neurons. The cells were fixed with 4% paraformaldehyde for 30 min and rinsed three times with PBS (Gibco BRL) for 5 min each. The cells were permeabilized with 0.2% Triton X-100/PBS for 5 min, blocked with 2% normal goat serum (NGS)/PBS for 1 h at room temperature, and then incubated overnight with following primary antibodies at 4 °C: 8-hydroxy-2′-deoxyguanosine (8-OHdG; 1:200; Santa Cruz Biotechnology, Dallas, TX, USA), nuclear respiratory factor 2 (Nrf2; 1:200; Abcam, Cambridge, UK), rhodamine phalloidin (F-actin; 1:1000; Invitrogen, Carlsbad, CA, USA), microtubule-associated protein 2 (MAP2; 1:1000; Synaptic Systems, Göttingen. Germany), neuron-specific class III beta-tubulin (Tuj1; 1:2000; R&D system), brain-derived neurotrophic factor (BDNF; 1:200; Abcam), nerve growth factor (NGF; 1:100; Abcam), Sirtuin1 (Sirt1; 1:1000; Cell Signaling Technology, Danvers, MA, USA), Sirtuin2 (Sirt2; 1:500; Sigma-Aldrich, Saint Louis, MO, USA) and synapsin1 (Syn1; 1:500; Synaptic Systems, Goettingen, Germany). After that, the samples were then incubated for 2 h with fluorescent secondary antibodies diluted at 1:300 in 2% NGS. The cells were washed and mounted with fluorescence mounting medium (Dako, Agilent Technologies, Santa Clara, CA, USA), and images of stained cortical neurons were captured by confocal microscopy (Eclipse C2 Plus, Nikon). Ten images were randomly taken at 10× or 40× objective with the identical parameters via confocal microscopy. Measuring intensity was performed using ImageJ software and compared quantitatively. And fluorescent signal intensity was quantified the intensity values occupied by the signal using ImageJ software (1.37v, National Institutes of Health, Bethesda, MD, USA).

### 2.6. Flow Cytometry

The cell-permeable fluorogenic probe, 2′, 7′-dichlorodihydrofluorescein diacetate (DCFDA; Sigma-Aldrich), which is a cell permeant reagent produced by oxidation of DCF by ROS within the cell, was used to evaluate the production of intracellular ROS. The 9.3 mg DCFDA powder was dissolved in high-quality 3.8 mL anhydrous dimethyl sulfoxide (Sigma-Aldrich), and then the pellet was suspended with 1 mL of 10 µM DCFDA solution. After further incubation at 37 °C for 30 min, a spectrofluorometer was used for measuring DCFDA fluorescence at 484 and 530 nm. The mean percentages of DCFDA-positive cells were determined relative to the control group.

### 2.7. DNA Dot-Blotting

A DNA dot-blotting was performed using extracted genomic DNA from a DNeasy Blood and Tissue Kit (QIAGEN, Hilden, Germany). The DNA concentration were measured by a microplate reader (Epoch, BioteK, Winooski, VT, USA) at 260 nm/280 nm absorbance. Purified DNA were loaded onto 0.2 µM nitrocellulose membrane and hybridized for 2 h at 80 °C. The membrane was then blocked with 5% skim milk in 1X Tris-buffered saline (Bio-Rad) with 0.1% Tween 20 (TBST; Sigma-Aldrich) at room temperature for 1 h. After incubation with dsDNA (1:2000, Abcam) and 8-OHdG (1:200, Santa Cruz Biotechnology, Dallas, TX, USA) at 4 °C for overnight, membrane was washed in three changes of TBST for 15 min and then incubated with an anti-mouse or anti-rabbit horseradish-peroxidase-conjugated antibody (1:2500, Abcam) at room temperature for 2 h. Dot blots were visualized using electrochemiluminescence (Bio-Rad, Hercules, CA, USA) and evaluated with an Amersham Imager 600 (GE Healthcare Life Sciences, Uppsala, Sweden).

### 2.8. Real-Time Polymerase Chain Reaction (PCR)

The changes in mRNA levels of *Sirt1*, *Sirt2*, *BDNF*, *NGF*, *GAP43*, and *NF200* were analyzed in each group using real-time PCR. Total RNA was isolated using TRIzol reagent (Ambion, Austin, TX, USA). cDNA was synthesized using oligo(dT)20 primer and Accupower RT PreMix (Bioneer, Daejeon, Korea). All primers are listed in Table 1. Real-time PCR was performed using iQ SYBR Green Supermix (Bio-Rad) on a CFX Connect Real-Time PCR Detection System (Bio-Rad). The cycling conditions were 40 cycles at 95 °C for 3 min, 95 °C for 15 s, and 60 °C for 30 s. All real-time PCR samples were evaluated in at least triplicate. The gene expression was normalized to that of GAPDH and is shown as the fold-change relative to the control group.

### 2.9. Western Blotting

Total proteins were isolated by lysis-centrifugation with RIPA buffer (CellNest, Minato, Tokyo, Japan), Protease Inhibitor Cocktail Set III (1:1000, Millipore, Billerica, MA, USA), and a Pierce BCA Protein Assay Kit (Thermo Fisher Scientific, Waltham, MA, USA) in accordance with the manufacturer’s protocol. Protein samples were separated by sodium dodecyl-sulfate polyacrylamide gel electrophoresis, transferred to a polyvinylidene difluoride membrane, blocked with 5% skim milk in 1X TBST and probed using various antibodies. The following antibodies were used: iNOS (500:1, R&D Systems, Minneapolis, MN, USA); HO-1 (1:500, Abcam); Nrf2 (1:500, Abcam); Sirt1 (1:1000, Cell Signaling Technology); Sirt2 (1:500, Sigma-Aldrich); β-actin (1:1000, Santa Cruz Biotechnology, Dallas, TX, USA); and subsequently incubated with horseradish peroxidase-conjugated anti-rabbit or anti-mouse antibodies (1:2500, Abcam). Signals were detected using electrochemiluminescence (Bio-Rad) and imaged with an Amersham Imager 600 (GE Healthcare Life Sciences, Uppsala, Sweden). Equivalent loading of protein was verified by probing for β-actin.

### 2.10. EX-527 Treatment

Cells were divided into five groups in the presence of EX-527 (Sirt1 inhibitor; Cayman Chemical, Ann Arbor, MI, USA) as follws; (1) EX-527, (2) EX-527+H_2_O_2_, (3) EX-527+GJD 10 µg/mL+H_2_O_2_, (4) EX-527+GJD 25 µg/mL+H_2_O_2,_ (5) EX-527+GJD 50 µg/mL+H_2_O_2_. Cells were pretreated with 10 μM EX-527 and GJD for 30 min before H_2_O_2_ treatment, and added with 30 μM H_2_O_2_.

### 2.11. Statistics

The standard error of the mean (SEM) was used for result expression. Statistical comparisons were performed using by one-way or two-way ANOVA with Tukey’s post-hoc analysis (Graph-Pad Prism 8.0, Inc., La Jolla, CA, USA). Significant differences were considered if the *p* value was ^#^ *p* < 0.05, ^##^ *p* < 0.01, ^###^ *p* < 0.001 and ^####^ *p* < 0.0001 vs. the blank group and * *p* < 0.05, ** *p* < 0.01, *** *p* < 0.001 and **** *p* < 0.0001 vs. the control group.

## 3. Results

### 3.1. GJD Exerted a Neuroprotective Effect on H_2_O_2_-Treated Cortical Neurons

**Figure 1:**
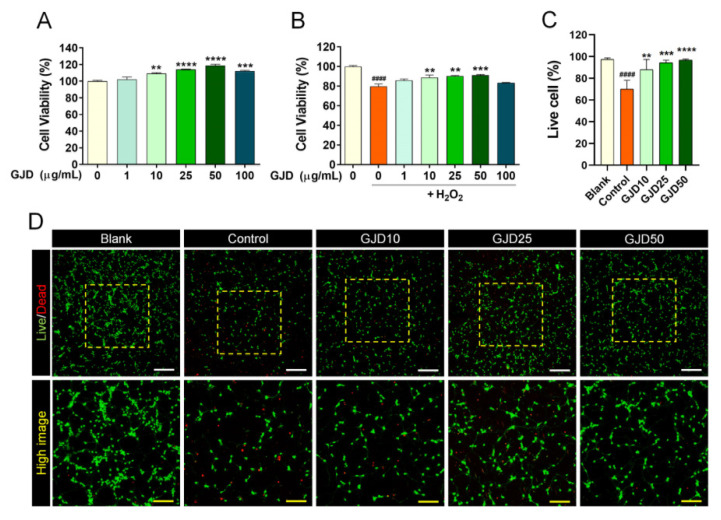
Neuronal viability of cortical neurons pretreated with GJD followed by subsequent exposure to H_2_O_2_ for 24 h. (**A**) Cell viability of primary cortical neurons treated with GJD (1, 10, 25, 50, and 100 µg/mL) for 24 h was determined using CCK-8 (*n* = 4). (**B**) Cell viability of primary cortical neurons pretreated with GJD (1, 10, 25, 50, and 100 µg/mL) followed with H_2_O_2_ (30 μM) was determined using CCK-8 (*n* = 4). (**C**) Number of green-stained live cells in each group (*n* = 4). Blank = non-treatment, Control = H_2_O_2_ only, GJD = GJD (µg/mL) + H_2_O_2_. (**D**) Representative images showing live (green) and dead (red) cells in the blank, control, and GJD groups. White scale bar = 200 μm, yellow scale bar = 100 μm. Data are expressed as the mean ± SEM. Significant differences indicated as ^####^ *p* < 0.0001 vs. blank group; ** *p* < 0.01, *** *p* < 0.001, and **** *p* < 0.0001 vs. control group were analyzed via ordinary one-way analysis of variance (ANOVA) with Tukey’s post hoc analysis.

We preliminarily determined the optimal doses of GJD to achieve therapeutic effects in H_2_O_2_-injured neuron using the CCK-8 assay. GJD was not cytotoxic to cortical neurons at doses of 1–100 µg/mL and significantly increased cell proliferation from 10 to 50 µg/mL (Figure 1A). Moreover, pretreatment with 10–50 µg/mL GJD effectively blocked cytotoxic activity and specifically exerted a significant neuroprotective effect on cortical neurons following H_2_O_2_ exposure (Figure 1B). We confirmed these optimal ranges of GJD for neuroprotective effect using imaging assays for live and dead cells. The number of green-stained living cells was significantly reduced after H_2_O_2_ treatment compared with the blank group. The number of live cells was significantly increased following GJD pretreatment, demonstrating that the cells were dose-dependently protected from H_2_O_2_ stress (Figure 1C,D). Based on the cell viability results, GJD treatment at up to 50 µg/mL was considered as safe in primary cortical neurons.

### 3.2. GJD Suppressed H_2_O_2_-Induced ROS Production by Activating Nrf2/HO-1 Expression in Cortical Neurons

**Figure 2:**
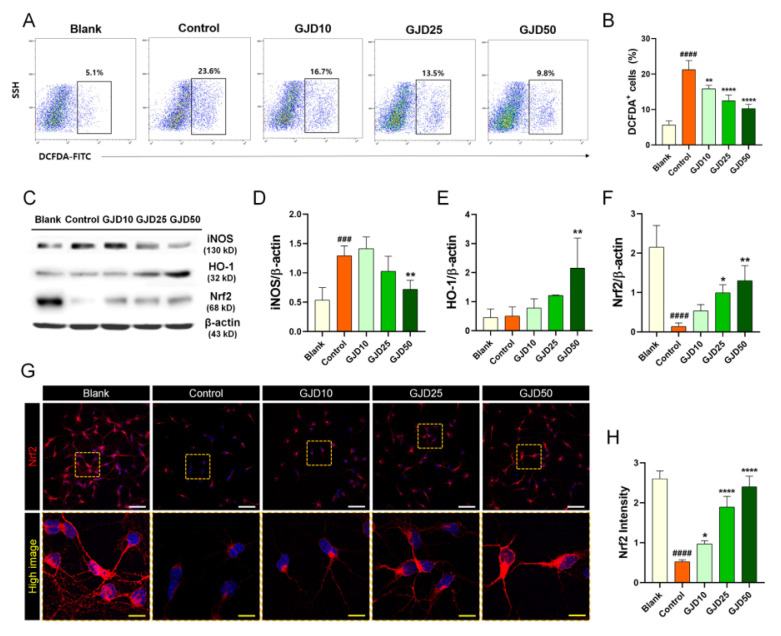
Antioxidant effect of cortical neurons pretreated with GJD in H_2_O_2_-injured neurons. (**A**) Representative flow cytometry plot images showing oxidized DCFDA for identifying ROS in blank, control, and GJD groups. (**B**) Quantitative flow cytometry data of DCFDA positivity in the blank, control, and GJD groups (*n* = 4). (**C**) Representative western blot results for cortical neurons with iNOS, Nrf2, and HO-1 in the blank, control, and GJD groups. (**D**–**F**) Relative quantification of the iNOS, HO-1, and Nrf2 protein expression in the blank, control, and GJD groups (*n* = 4). (**G**) Representative immunofluorescent images of Nrf2 (red) in cortical neurons of the blank, control, and GJD groups. White scale bar = 50 μm, yellow scale bar = 10 μm. (**H**) Quantification of Nrf2 intensity in the blank, control, and GJD groups (*n* = 5). Data are expressed as the mean ± SEM. Significant differences indicated as ^###^ *p* < 0.001, ^####^ *p* < 0.0001 vs. blank group; * *p* < 0.05, ** *p* < 0.01, and **** *p* < 0.0001 vs. control group were analyzed via one-way ANOVA with Tukey’s post hoc analysis.

The major consequence of oxidative stress is the induction of DNA damage along with increased intracellular ROS. To determine whether the GJD could prevent DNA damage caused by H_2_O_2_-induced oxidative stress, we used three methods to evaluate ROS and 8-OHdG expression. First, we measured intracellular ROS levels using H2DCF-DA as a good indicator of cellular ROS by flow-cytometry. The ROS level was dramatically increased following exposure of the neurons to H_2_O_2_ but was dose-dependently decreased following pretreatment with GJD (Figure 2A). Changes in cellular ROS levels were precisely measured by quantifying the percentage of H2DCF-DA positivity. The cells were detected in approximately 23% of 10,000 single cells after H_2_O_2_ treatment. Additionally, H2DCF-DA-positive cells showed a dose-dependent decrease after GJD pretreatment, and a significant decrease compared with the control group (Figure 2B). We next investigated iNOS, HO-1, and Nrf2 expression to confirm whether GJD inhibits oxidative stress-related iNOS activity by modulating Nrf2/HO-1 activation. The iNOS level increased with H_2_O_2_ treatment, whereas this level gradually decreased in the 25 and 50 µg/mL GJD groups (Figure 2C,D). In accordance with previous studies, activating the antioxidant protein HO-1 and nuclear factor erythroid derived 2-related factor 2 (Nrf2) play crucial regulatory roles in the cellular stress response and neuroprotection [26]. Thus, we also performed Western blotting and immunochemical staining to confirm whether GJD could induce the expression of Nrf2/HO-1 in cortical neurons against H_2_O_2_-induced neurotoxicity. After H_2_O_2_ treatment, the level of HO-1 protein was not increased in control group; however, the HO-1 protein level showed a tendency to increase in a concentration-dependent manner with GJD pretreatment, as shown in Figure 2C. Quantitative analysis revealed significant increase in 50 µg/mL GJD group compared to control group (Figure 2E). We also analyzed the protein expression of Nrf2 and confirmed the antioxidant activity and prevention of oxidative damage by GJD. As shown in Figure 2C,F, the Western blot assay revealed that 25 and 50 µg/mL GJD groups significantly increased Nrf2 level compared to control group. Confocal images also showed that the Nrf2 level significantly decreased after H_2_O_2_ treatment compared to that in the blank group, with GJD affecting Nrf2 expression in a dose-dependent manner, supporting its antioxidant action (Figure 2G,H).

### 3.3. GJD Prevented Oxidative Damage to DNA in H_2_O_2_-Treated Cortical Neurons

**Figure 3:**
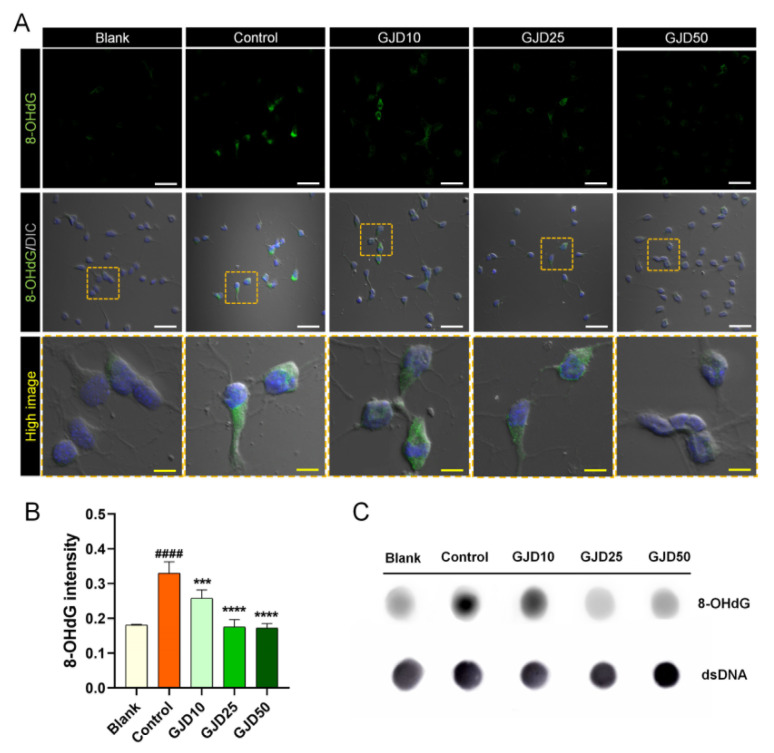
Preventive effect of cortical neurons pretreated with GJD against H_2_O_2_-induced oxidative DNA damage. (**A**) Representative immunofluorescence and differential interference contrast merged images of 8-OHdG (green) in cortical neurons of the blank, control, and GJD groups. White scale bar = 50 μm, yellow scale bar = 10 μm. (**B**) Quantification of 8-OHdG intensity in the blank, control, and GJD groups (*n* = 5). (**C**) Representative dot blot results of 8-OHdG expression for 500 ng of genomic DNA in the blank, control, and GJD groups. Data are expressed as the mean ± SEM. Significant differences indicated as ^####^ *p* < 0.0001 compared vs. blank group; *** *p* < 0.001 and **** *p* < 0.0001 vs. control group were analyzed via ordinary one-way ANOVA with Tukey’s post hoc analysis.

Next, we confirmed the dose-dependent preventive effect of GJD against DNA damage. 8-OHdG is used widely as a reliable marker of oxidative DNA damage and stress [27]. The 8-OHdG level was first determined using immunochemical staining and a dot-blot assay. GJD pretreatment reduced the 8-OHdG expression increased by H_2_O_2_ (Figure 3A). There was significantly greater difference between the control and GJD groups with respect to the 8-OHdG intensity. H_2_O_2_ treatment induced positively stained cells with 8-OHdG; however, the intensity of 8-OHdG-positive neurons significantly decreased in a dose-dependent manner by GJD pretreatment (Figure 3B). We also assessed the 8-OHdG level in genomic DNA using a dot blot assay (Figure 3C). The 8-OHdG level in genomic DNA was markedly decreased in the GJD groups compared to the control group. These findings indicate that GJD inhibits H_2_O_2_-induced DNA damage in cortical neurons.

### 3.4. GJD Activated Sirt1 Expression for Axonal Outgrowth but Did Not Affect Sirt2 Expression in H_2_O_2_-Induced Cortical Neurons

**Figure 4:**
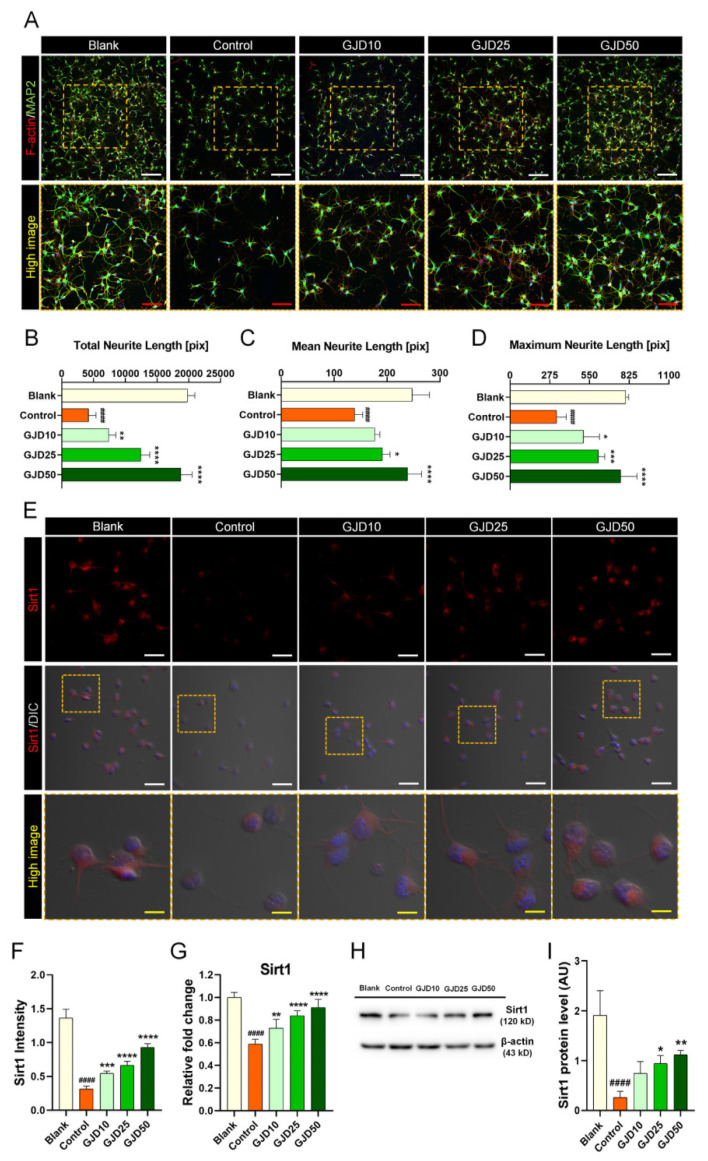
Promotion of axonal outgrowth by activating Sirt1 signaling pathway in cortical neurons pretreated with GJD against H_2_O_2_ stress. (**A**) Immunofluorescent images of MAP2 (green) and F-actin (red) in cortical neurons of the blank, control, and GJD groups. White scale bar = 200 μm, red scale bar = 100 μm. (**B**–**D**) Quantification of axonal outgrowth by three parameters in the blank, control, and GJD groups (*n* = 5). (**E**) Representative immunofluorescence and differential interference contrast merged images of Sirt1 (red) in cortical neurons of the blank, control, and GJD groups. White scale bar = 50 μm, yellow scale bar = 10 μm. (**F**) Quantification of Sirt1 intensity in the blank, control, and GJD groups (*n* = 5). (**G**) Relative fold-changes in the Sirt1 mRNA level in the blank, control, and GJD groups (*n* = 5). (**H**) Representative western blot band for Sirt1 in the blank, control, and GJD groups. (**I**) Relative quantification of the Sirt1 protein expression in the blank, control, and GJD groups (*n* = 4). Data are expressed as the mean ± SEM. Significant differences indicated as ^####^ *p* < 0.0001 compared vs. blank group; * *p* < 0.05, ** *p* < 0.01, *** *p* < 0.001, and **** *p* < 0.0001 vs. control group were analyzed via ordinary one-way ANOVA with Tukey’s post hoc analysis.

We further assessed the promoting effect of GJD on axonal outgrowth in H_2_O_2_-treated neurons. Sirt1 was previously reported to be involved in the processes of neuron survival, outgrowth, and synaptic plasticity [28]. Thus, we examined the effect of GJD on the promotion of neurite outgrowth by activating the Sirt1 signaling pathway. GJD pretreatment not only protected cortical neuron against H_2_O_2_ damage, but also dose-dependently increased neurite outgrowth. A significant increase in MAP2-positive neurite was observed in GJD-pretreated neurons (Figure 4A). When axon outgrowth was quantified by three parameters, the results revealed a significant dose-response effect in neurons pretreated with GJD against H_2_O_2_-induced injury (Figure 4B–D). Additionally, F-actin assembly was observed at the leading edge to the axons. We next examined whether GJD affected Sirtuin expression to accelerate axonal growth in H_2_O_2_-treated neurons. Almost all neurons strongly expressed Sirt1 before H_2_O_2_ exposure, whereas Sirt1 was rarely expressed following H_2_O_2_ treatment. Thus, Sirt1 was activated by GJD pretreatment to promote axonal growth (Figure 4E), and its intensity was significantly elevated in a dose-dependent manner compared to that in the control group (Figure 4F). In addition, the mRNA and protein expression of Sirt1 was investigated using real-time PCR and Western blot. GJD pretreatment induced a significant dose-dependent increase in Sirt1 expression, which showed a dose-related increasing trend in GJD-pretreated neurons as directly observed in the confocal images (Figure 4G,H). Quantifying the Western blot exhibited a significant increase of Sirt1 level in 25 and 50 µg/mL GJD groups compared to the control group (Figure 4I). We also analyzed the Sirt2 level using the same methods as in Sirt1 analysis. Sirt2 expression was also decreased by H_2_O_2_ treatment, but GJD did not affect Sir2 expression in H_2_O_2_-treated neurons. Confocal images revealed that Sirt2 expression was decreased after H_2_O_2_ treatment, but GJD did not stimulate a significant increase in H_2_O_2_-treated neurons (Supplementary Figure S1A). Sirt2 intensity was decreased by H_2_O_2_ exposure, but there was no significant difference between the control and GJD groups (Supplementary Figure S1B). This trend was also observed in the real-time PCR and Western blotting results (Supplementary Figure S1C,D). Quantifying data generated by Western blot revealed significant reduction in control group, compared to blank group. However, GJD pretreatment does not induce increased expression of Sirt2 level (Supplementary Figure S1E). Based on these findings, GJD can stimulate axonal outgrowth by increasing Sirt1 expression but does not affect Sirt2 expression in H_2_O_2_-injured neurons.

### 3.5. GJD Did Not Induce Axonal Growth in Sirt1-Silenced Neurons

**Figure 5:**
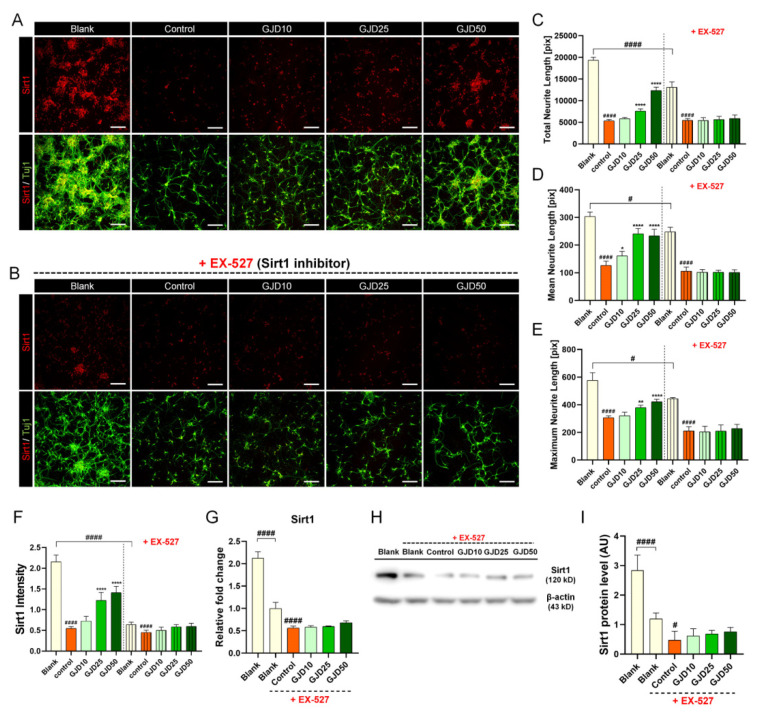
Effect on neurite outgrowth of cortical neurons treated with GJD and Sirt1 inhibitor EX-527 with H_2_O_2_ exposure. (**A**) Immunofluorescence images of Sirt1 (red) and Tuj1 (green) in primary cortical neurons of the blank, control, and GJD groups. (**B**) Immunofluorescence images of Sirt1 (red) and Tuj1 (green) in primary cortical neurons of the blank, control, and GJD groups under Sirt1-inhibited condition. White scale bar = 200 μm. (**C**–**E**) Quantification of axonal outgrowth with or without EX-527 using three parameters in the blank, control, and GJD groups (*n* = 5). (**F**) Quantification of Sirt1 intensity with or without EX-527 in the blank, control, and GJD groups (*n* = 5). (**G**) Relative fold-changes in the Sirt1 mRNA level with EX-527 in the blank, control, and GJD groups (*n* = 5). (**H**) Representative Western blot band for Sirt1 with EX-527 in the blank, control, and GJD groups. (**I**) Relative quantification of the Sirt1 protein expression with EX-527 in the blank, control, and GJD groups (n = 4). Data are expressed as the mean ± SEM of three separate experiments performed in duplicate. Significant differences indicated as ^#^ *p* < 0.05, ^####^ *p* < 0.0001 compared vs. blank group; * *p* < 0.05, ** *p* < 0.01, and **** *p* < 0.0001 vs. control group were analyzed via ordinary one-way ANOVA with Tukey’s post hoc analysis.

Additionally, we tested whether GJD can induce axon growth when the Sirt1 pathway was inhibited by the specific Sirt1 inhibitor EX-527 to better understand the relevance of Sirt1 activation more clearly. In our work, we confirmed that Sirt1 expression decreased after H_2_O_2_ treatment, while increased with axonal elongation in GJD-pretreated conditions (Figure 5A), indicating that Sirt1 may be related to the neuroprotective and axon growth-promoting effect of GJD pretreatment. Surprisingly, after adding a Sirt1 inhibitor EX-527, we found that neuronal death was increased and cell viability was decreased, confirming that Sirt1 was involved in neuronal survival. Next, when adding both GJD and EX-527 in H_2_O_2_-injured neuron, axonal outgrowth was not induced even if GJD was pretreated in the neuron before H_2_O_2_ exposure. All dose-dependent effects of GJD pretreatment were abrogated by the Sirt1 inhibitor EX-527 (Figure 5B). Therefore, we demonstrated that Sirt1 may be closely related to the neuroprotective effect of GJD, suggesting its dependency on the Sirt1 signaling pathway. Moreover, axon growth showed no obvious difference either with or without GJD under the Sirt1-inhibited condition (Figure 5C–E), indicating its great potential pathway for the preventive effect of GJD. In addition, the Sirt1 intensity did not show any difference between GJD and control groups under the Sirt1-inhibited condition (Figure 5F). These Sirt1 expression tend to have similar patterns at the RNA and protein levels. Under condition of EX-527 exposure, there was no significant overall difference between GJD and control groups (Figure 5G–I).

### 3.6. GJD Influenced Neurotrophic Factor Upregulation in H_2_O_2_-Damaged Cortical Neurons

**Figure 6:**
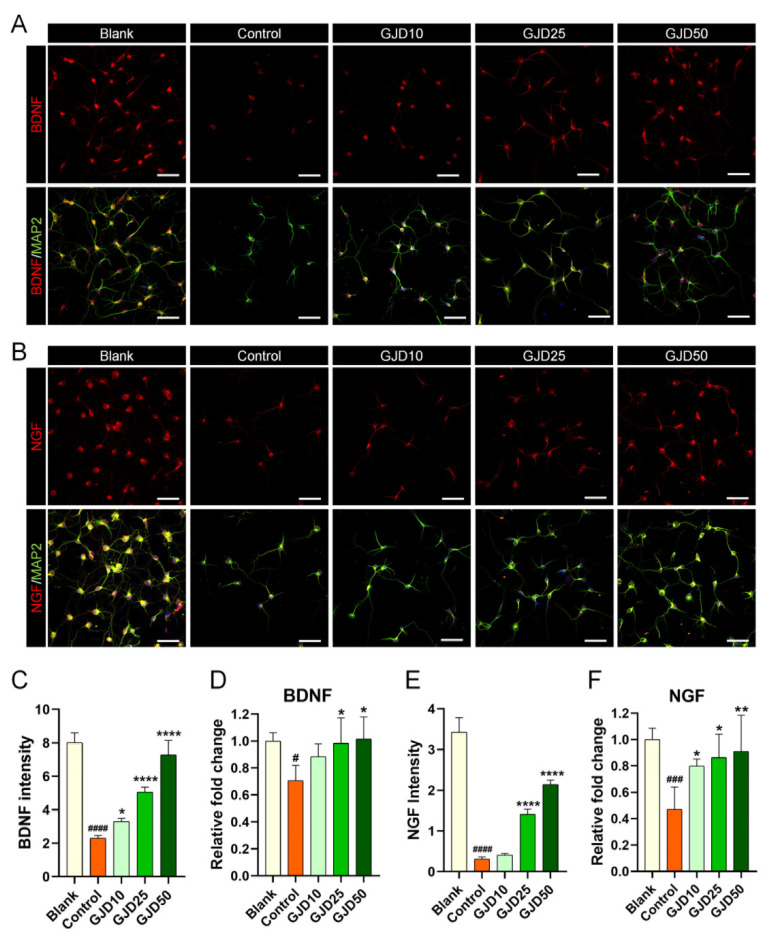
Neurotrophic effect on cortical neurons pretreated with GJD against H_2_O_2_-induced damage. (**A**,**B**) Representative immunofluorescent images of MAP2 (green) with BDNF or NGF (red) in cortical neurons of the blank, control, and GJD groups. White scale bar = 50 μm. (**C**) Quantification of BDNF intensity in the blank, control, and GJD groups (*n* = 5). (**D**) Relative fold-changes in the level of BDNF mRNA in the blank, control, and GJD groups (*n* = 5). (**E**,**F**) Quantification of NGF intensity and mRNA in the blank, control, and GJD groups (n = 5). Data are expressed as the means ± SEM. Significant differences indicated as ^#^ *p* < 0.05, ^###^ *p* < 0.001, and ^####^ *p* < 0.0001 compared vs. blank group; * *p* < 0.05, ** *p* < 0.01 and **** *p* < 0.0001 vs. control group were analyzed via ordinary one-way ANOVA with Tukey’s post hoc analysis.

Based on the results highlighting the effect of GJD in mediating the Sirt1 level, we also investigated neurotrophic factor expression to better understand how GJD can accelerate axon growth by increasing Sirt1 expression. Previous studies demonstrated that Sirt1 acts as a neuroprotective regulator by activating BDNF and NGF expression, and neurotrophic factors are well-known to be essential for neuronal survival and growth by participating in synaptic function and plasticity [29,30]. Confocal microscopy images revealed that BDNF expression was decreased after H_2_O_2_ treatment but dose-dependently increased in the GJD groups (Figure 6A). Quantitative analysis of BDNF showed that the intensity was significantly higher in the GJD groups than in the control group (Figure 6C). In addition, the BDNF mRNA level, analyzed by real-time PCR, was significantly upregulated in the 25 and 50 µg/mL GJD groups compared to that in the control group (Figure 6D). A similar trend was observed in the confocal images of NGF, as BDNF increased from GJD pretreatment and promoted axonal growth in H_2_O_2_-injured neurons (Figure 6B). The NGF intensity was significantly increased in 25 and 50 µg/mL GJD groups (Figure 6E). The NGF mRNA level had significant upregulation in the 25 and 50 µg/mL GJD groups compared to that in the control group (Figure 6F). These findings suggest that pretreatment with GJD can promote axonal growth by activating BDNF and NGF, which involves the Sirt1 signaling pathway, in H_2_O_2_-injured neurons.

### 3.7. GJD Enhanced Synapse Formation by Upregulating Synasin1 Expression in H_2_O_2_-Induced Cortical Neurons

**Figure 7:**
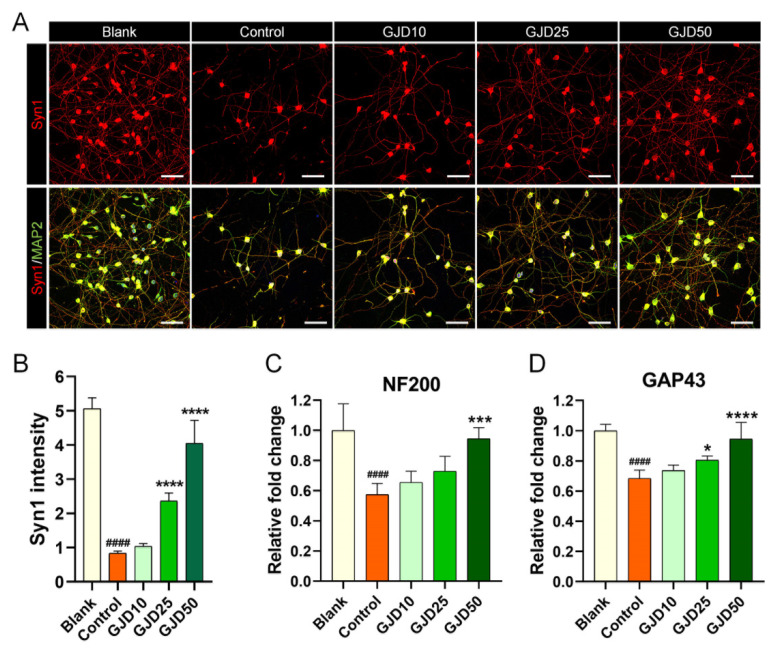
Synapse formation effect of cortical neurons pretreated with GJD against H_2_O_2_-induced synaptic loss. (**A**) Representative immunofluorescence images of MAP2 (green) with Syn1 (red) in cortical neurons of the blank, control, and GJD groups. White scale bar = 50 μm. (**B**) Quantification of Syn1 intensity in the blank, control, and GJD groups (*n* = 5). (**C**,**D**) Relative fold-changes in the level of NF200 and GAP43 mRNA in the blank, control, and GJD groups (*n* = 5). Data are expressed as the mean ± SEM. Significant differences indicated as ^####^ *p* < 0.0001 compared vs. blank group; * *p* < 0.05, *** *p* < 0.001 and **** *p* < 0.0001 vs. control group were analyzed via ordinary one-way ANOVA with Tukey’s post-hoc analysis.

Synapse contacts are typically formed between pre-and postsynaptic cells to ensure the normal function of neural networks. Remarkably, during injury to the central nervous system, synapse loss occurs almost simultaneously in billions of neurons, resulting in permanent disability. Thus, to determine whether GJD can lead to synapse formation in H_2_O_2_-injured neurons, Syn1 staining was performed to confirm the synaptic contacts induced by GJD pretreatment. The signal for Syn1 was bright between neurons within the cell soma and axons. Meanwhile, this signal was dramatically decreased after H_2_O_2_ exposure. The GJD-pretreated condition induced an increase in the Syn1 signal in a dose-dependent manner (Figure 7A), with significant increases in the 25 and 50 µg/mL GJD and control groups (Figure 7B). Furthermore, neurofilament 200-kDa (NF200) and growth-associated protein (GAP43) were enhanced by GJD pretreatment. Quantitatively, GJD induced a dose-dependent increase in NF200 expression, but only 50 µg/mL GJD showed a significant effect (Figure 7C). GAP43 mRNA level was also dose-dependently increased and significantly different in the 25 and 50 µg/mL GJD groups relative to the control group (Figure 7D).

## 4. Discussion

Although GJD has been demonstrated to have neuroprotective effects, its mechanism in the repair and prevention of neurological damage has not been widely studied. Thus, we examined whether GJD can protect the neurons from H_2_O_2_-induced oxidative damage by activating the Sirt1 signaling pathway. Sirt1 is involved in numerous biological and pathophysiological processes and has recently been considered as an emerging neuronal therapeutic target for neurological diseases [31]. The GJD used in this study showed excellent in vitro activity against H_2_O_2_-induced oxidative stress; we demonstrated these specific effects directly in primary cortical neurons. GJD was highly effective for protecting against neuronal death caused by oxidative stress. Here, we found that 10–50 µg/mL GJD protected primary cortical neurons; these concentrations are recommended as the optimal therapeutic dose range for achieving antioxidant neuroprotective effects.

Previous studies also showed that GJD enhanced neurite outgrowth in neuronal-like cell lines, PC12 cells and increased NGF secretion in astrocytes treated at 250 µg/mL [17]. Although the therapeutic dose range of GJD was previously suggested by Moon et al., the authors did not directly demonstrate these effects in neural cells with the same morphological and biochemical characteristics as mature neurons. We also demonstrated that GJD has excellent antioxidant activity by reducing the ROS level following H_2_O_2_ exposure in cortical neurons and specifically prevented oxidative DNA damage. One important feature of oxidative stress is the accumulation of oxidative damage to DNA, RNA, protein, and organelles [32]. GJD prevents oxidative stress-induced DNA damage and promotes neurite outgrowth under H_2_O_2_-treated conditions. Specifically, axon growth was accelerated through the Sirt1 signaling pathway following GJD treatment. Sirt1 was dose-dependently upregulated and peaked at 50 µg/mL of GJD, and concomitantly induced axon elongation from oxidative damage. In contrast, Sirt2 expression was not elevated by GJD treatment in H_2_O_2_-treated neurons.

Additionally, we examined whether GJD could induce axon growth when Sirt1 pathway was inhibited by inhibitor to confirm the relevance of Sirt1 activation. Sirt1 inhibition by EX-527 did not robustly induce axon growth in neurons, even following GJD treatment. This result indicates that the preventive neuroprotection effect of GJD is closely associated with the Sirt1 signaling pathway in neurons following oxidative injury. Cumulative evidence regarding the mechanisms involved in Sirt1-induced neuroprotection indicates that Sirt1 activation can directly control Forkhead box protein 1 to defense oxidative stress, NF-кB to anti-inflammation, and p53 to anti-apoptosis and providing effective neuroprotection against neurological diseases [33,34]. Therefore, the Sirt1 activation effect of GJD may represent a therapeutic target for future research on anti-aging and immune regulation.

One limitation of our study was that it was difficult to identify a single target responsible for the neuroprotective properties and mechanism of action of GJD, which is a complex multi-herbal formula. Another limitation was that interpreting functional recovery at the tissue level was difficult. Thus, studies are needed to investigate the preventive effect of GJD in an animal model and human-induced cell line. Our results reveal the antioxidant neuroprotective effects of GJD in H_2_O_2_-injured mature cortical neurons, and that the Sirt1 signaling pathway is involved in the mechanism of action of GJD.

## 5. Conclusions

GJD enhances the axonal outgrowth of cortical neurons through Sirt1 regulation following H_2_O_2_-induced oxidative injury.

## Figures and Tables

**Table 1:** Primer sequences used for real-time polymerase chain reaction (PCR) analysis.

Gene	5′-3′	Primer Sequence
*Sirt1*	Forward	AGGGAACCTCTGCCTCATCT
	Reverse	TGGCATACTCGCCACCTAAC
*Sirt2*	Forward	GGCGGCATGGATTTTGACTC
	Reverse	TTCCAGCTCCTTCCATCCCA
*BDNF*	Forward	CTTGGAGAAGGAAACCGCCT
	Reverse	GTCCACACAAAGCTCTCGGA
*NGF*	Forward	CCAAGGACGCAGCTTTCTATC
	Reverse	CTGTGTCAAGGGAATGCTGAAG
*GAP43*	Forward	TGCCCTTTCTCAGATCCACT
	Reverse	TTGCCACACAGAGAGAGAGG
*NF200*	Forward	AACACCACTTAGATGGCGGG
	Reverse	ACGTGGAGCGTTCAGCAATA
*GAPDH*	Forward	CCCCCAATGTATCCGTTGTG
	Reverse	TAGCCCAGGATGCCCTTTAGT

*Sirt1:* Sirtuin1; *Sirt2*: Sirtuin2; *BDNF*: brain-derived neurotrophic factor; *NGF*: nerve growth factor; *GAP43*: growth-associated protein 43; *NF200*: neurofilament 200; *GAPDH*: glyceraldehyde 3-phosphate dehydrogenase.

## Data Availability

The data presented in this study are available upon request from the corresponding author.

## Supplementary Materials

The following are available online at www.mdpi.com/article/10.3390/nu13124290/s1, Figure S1: Sirt2 analysis of cortical neuron pretreated with 10, 25, and 50 µg/mL of GJD with H_2_O_2_ exposure.

## References

[1] Chen X., Guo C., Kong J. (2012). Oxidative stress in neurodegenerative diseases. Neural Regen. Res..

[2] Singh A., Kukreti R., Saso L., Kukreti S. (2019). Oxidative stress: A key modulator in neurodegenerative diseases. Molecules.

[3] Kurutas E.B. (2016). The importance of antioxidants which play the role in cellular response against oxidative/nitrosative stress: Current state. Nutr. J..

[4] Birben E., Sahiner U.M., Sackesen C., Erzurum S., Kalayci O. (2012). Oxidative stress and antioxidant defense. World Allergy Organ. J..

[5] Agnihotri A., Aruoma O.I. (2020). Alzheimer’s disease and Parkinson’s disease: A nutritional toxicology perspective of the impact of oxidative stress, mitochondrial dysfunction, nutrigenomics and environmental chemicals. J. Am. Coll. Nutr..

[6] Kim G.H., Kim J.E., Rhie S.J., Yoon S. (2015). The role of oxidative stress in neurodegenerative diseases. Exp. Neurobiol..

[7] Cornelius C., Crupi R., Calabrese V., Graziano A., Milone P., Pennisi G., Radak Z., Calabrese E.J., Cuzzocrea S. (2013). Traumatic brain injury: Oxidative stress and neuroprotection. Antioxid. Redox Signal..

[8] Jia Z., Zhu H., Li J., Wang X., Misra H., Li Y. (2011). Oxidative stress in spinal cord injury and antioxidant-based intervention. Spinal Cord.

[9] Lee J.H., Jo D.C., Kim C.G., Moon S.J., Park T.Y., Ko Y.S., Song Y.S., Lee J.H. (2013). A literature review of effectiveness on the Gongjin-dan (Gongchen-dan). J. Korean Med. Rehabil..

[10] Kiyama R. (2017). DNA microarray-based screening and characterization of traditional chinese medicine. Microarrays.

[11] Rho S., Kang M., Choi B., Sim D., Lee J., Lee E., Cho C., Oh J.-W., Park S., Ko S. (2005). Effects of Yukmijihwang-tang derivatives (YMJd), a memory enhancing herbal extract, on the gene-expression profile in the rat hippocampus. Biol. Pharm. Bull..

[12] Son M.J., Im H.-J., Kim Y.-E., Ku B., Lee J.-H., Son C.-G. (2016). Evaluation of the anti-fatigue effects of a traditional herbal drug, Gongjin-dan, under insufficient sleep conditions: Study protocol for a randomised controlled trial. Trials.

[13] Son M.J., Im H.-J., Ku B., Lee J.-H., Jung S.Y., Kim Y.-E., Lee S.B., Kim J.Y., Son C.-G. (2018). An herbal drug, Gongjin-dan, ameliorates acute fatigue caused by short-term sleep-deprivation: A randomized, double-blinded, placebo-controlled, crossover clinical trial. Front. Pharmacol..

[14] Hong S.-S., Lee J.-Y., Lee J.-S., Lee H.-W., Kim H.-G., Lee S.-K., Park B.-K., Son C.-G. (2015). The traditional drug Gongjin-dan ameliorates chronic fatigue in a forced-stress mouse exercise model. J. Ethnopharmacol..

[15] Jung J.-W., Jeon S.H., Bae W.J., Kim S.J., Chung M.S., Yoon B.I., Choi S.W., Ha U.S., Hwang S.Y., Kim S.W. (2017). Suppression of oxidative stress of modified Gongjin-dan (WSY-1075) in detrusor underactivity rat model bladder outlet induced by obstruction. Chin. J. Integr. Med..

[16] Lee J.-S., Hong S.-S., Kim H.-G., Lee H.-W., Kim W.-Y., Lee S.-K., Son C.-G. (2016). Gongjin-dan enhances hippocampal memory in a mouse model of scopolamine-induced amnesia. PLoS ONE.

[17] Moon E., Her Y., Lee J.B., Park J.-H., Lee E.H., Kim S.-H., Oh M.S., Jang C.-G., Kim S.Y. (2009). The multi-herbal medicine Gongjin-dan enhances memory and learning tasks via NGF regulation. Neurosci. Lett..

[18] Sunwoo Y.-Y., Park S.I., Chung Y.-A., Lee J., Park M.-S., Jang K.-S., Maeng L.-S., Jang D.-K., Im R., Jung Y.J. (2012). A pilot study for the neuroprotective effect of Gongjin-dan on transient middle cerebral artery occlusion-induced ischemic rat brain. Evid.-Based Complement. Altern. Med..

[19] Li Y., Xu W., McBurney M.W., Longo V.D. (2008). SirT1 inhibition reduces IGF-I/IRS-2/Ras/ERK1/2 signaling and protects neurons. Cell Metab..

[20] Song W., Liu M.-L., Zhao Z.-J., Huang C.-Q., Xu J.-W., Wang A.-Q., Li P., Fan Y.-B. (2020). SIRT1 inhibits high shear stress-induced apoptosis in rat cortical neurons. Cell. Mol. Bioeng..

[21] Xu J., Jackson C.W., Khoury N., Escobar I., Perez-Pinzon M.A. (2018). Brain SIRT1 mediates metabolic homeostasis and neuroprotection. Front. Endocrinol..

[22] Zhang X.-S., Wu Q., Wu L.-Y., Ye Z.-N., Jiang T.-W., Ling-Yun W., Zhuang Z., Zhou M.-L., Zhang X., Hang C.-H. (2016). Sirtuin 1 activation protects against early brain injury after experimental subarachnoid hemorrhage in rats. Cell Death Dis..

[23] Olmos Y., Gomez F.S., Wild B., García N., Cabezudo S., Lamas S., Monsalve M. (2013). SirT1 regulation of antioxidant genes is dependent on the formation of a FoxO3a/PGC-1α complex. Antioxid. Redox Signal..

[24] Elibol B., Kilic U. (2018). High levels of SIRT1 expression as a protective mechanism against disease-related conditions. Front. Endocrinol..

[25] Kim H., Hong J.Y., Jeon W.-J., Lee J., Baek S.H., Ha I.-H. (2021). *Lycopus lucidus* turcz exerts neuroprotective effects against H_2_O_2_-induced neuroinflammation by inhibiting NLRP3 inflammasome activation in cortical neurons. J. Inflamm. Res..

[26] Loboda A., Damulewicz M., Pyza E., Jozkowicz A., Dulak J. (2016). Role of Nrf_2_/HO_−1_ system in development, oxidative stress response and diseases: An evolutionarily conserved mechanism. Cell. Mol. Life Sci..

[27] Valavanidis A., Vlachogianni T., Fiotakis C. (2009). 8-hydroxy-2′-deoxyguanosine (8-OHdG): A critical biomarker of oxidative stress and carcinogenesis. J. Environ. Sci. Health Part. C.

[28] Guo W., Qian L., Zhang J., Zhang W., Morrison A., Hayes P., Wilson S., Chen T., Zhao J. (2011). Sirt1 overexpression in neurons promotes neurite outgrowth and cell survival through inhibition of the mTOR signaling. J. Neurosci. Res..

[29] Herskovits A.Z., Guarente L. (2014). SIRT1 in neurodevelopment and brain senescence. Neuron.

[30] Shen J., Xu L., Qu C., Sun H., Zhang J. (2018). Resveratrol prevents cognitive deficits induced by chronic unpredictable mild stress: Sirt1/miR-134 signalling pathway regulates CREB/BDNF expression in hippocampus in vivo and in vitro. Behav. Brain Res..

[31] Yang Q., Zhou Y., Sun Y., Luo Y., Shen Y., Shao A. (2020). Will sirtuins be promising therapeutic targets for TBI and associated neurodegenerative diseases?. Front. Neurosci..

[32] Hong J., Kim H., Jeon W.-J., Lee J., Ha I.-H. (2021). Elevated mitochondrial reactive oxygen species within cerebrospinal fluid as new index in the early detection of lumbar spinal stenosis. Diagnostics.

[33] Khan M., Ullah R., Rehman S.U., Shah S.A., Saeed K., Muhammad T., Park H.Y., Jo M.H., Choe K., Rutten B.P.F. (2019). 17beta-estradiol modulates SIRT1 and halts oxidative stress-mediated cognitive impairment in a male aging mouse model. Cells.

[34] Zhang F., Wang S., Gan L., Vosler P.S., Gao Y., Zigmond M.J., Chen J. (2011). Protective effects and mechanisms of sirtuins in the nervous system. Prog. Neurobiol..

