# Supplementary material for: Gongjin-Dan Enhances Neurite Outgrowth of Cortical Neuron by Ameliorating H2O2-Induced Oxidative Damage via Sirtuin1 Signaling Pathway
Source: Nutrients. 2021 Nov 27;13(12):4290. doi: 10.3390/nu13124290 (PMC8707945; doi:10.3390/nu13124290)
Supplement: Supplementary file 1 [file nutrients-13-04290-s001.zip › nutrients-1472086-SI.pdf]

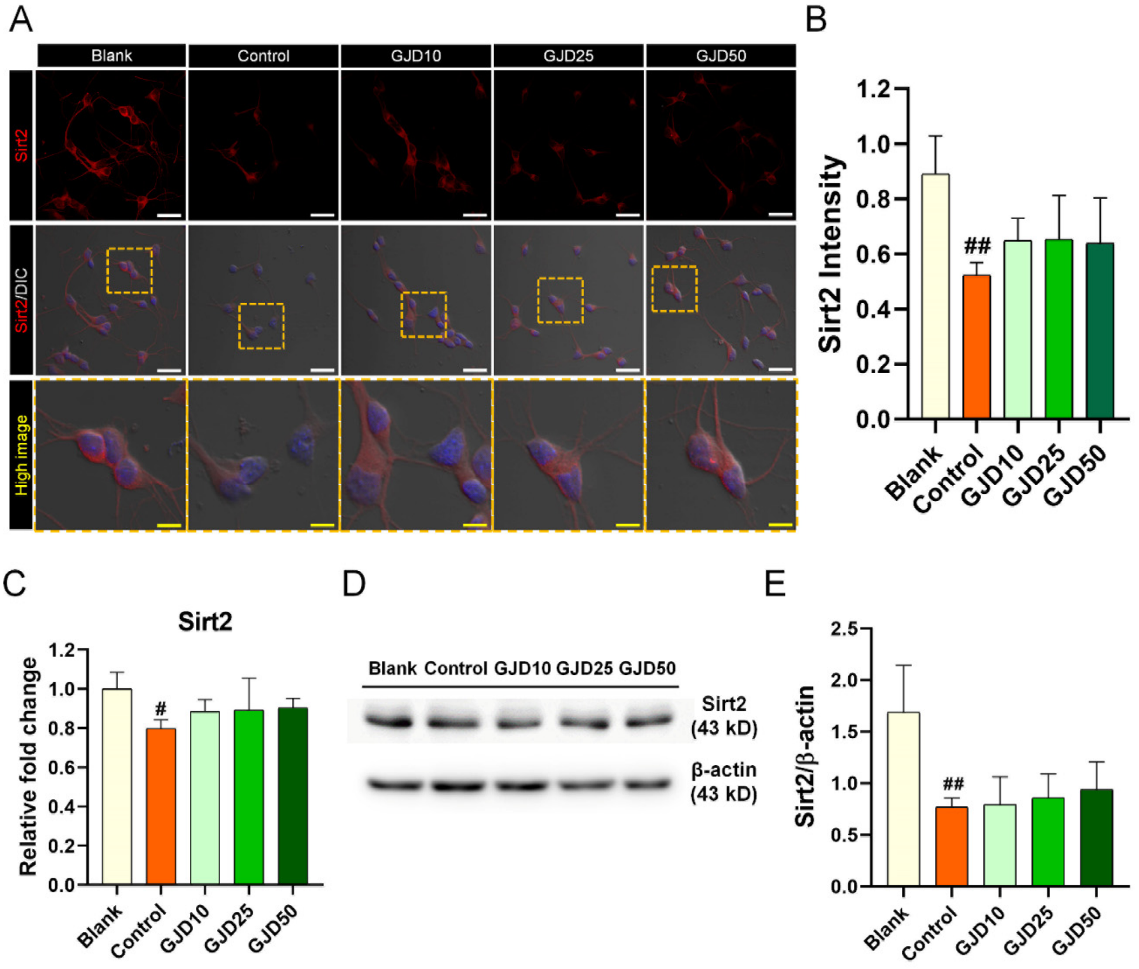

Figure S1. Effect on Sirt2 signaling pathway of cortical neurons pretreated with GJD with H_2O_2 exposure. (A) Representative immunofluorescence and differential interference contrast merged images of Sirt2 (red) in cortical neurons of the blank, control, and GJD groups. White scale bar= 50 µm, Yellow scale bar= 10 µm. (B) Quantification of Sirt2 intensity in the blank, control, and GJD groups (n= 5). (C) Relative fold-changes in the Sirt2 mRNA level in the blank, control, and GJD groups (n= 5). (D) Representative western blot band for Sirt2 in the blank, control, and GJD groups. (E) Relative quantification of the Sirt2 protein expression in the blank, control, and GJD groups (n=4). Data are expressed as the mean ± SEM. Significant differences indicated as $^{\#}p < 0.05$, $^{\#\#}p < 0.01$ compared vs. blank group were analyzed via ordinary one-way ANOVA with Tukey's post hoc analysis.
